# The complete mitochondrial genome of a burying beetle, *Nicrophorus nepalensis* Hope, 1831 (Coleoptera: Silphidae)

**DOI:** 10.1080/23802359.2021.1930220

**Published:** 2021-05-24

**Authors:** Yanpeng Cai, Xiaoyan Li

**Affiliations:** a School of Basic Medicine, Guizhou University of Traditional Chinese Medicine, Guiyang, China; b Hebei Key Laboratory of Animal Diversity, Langfang Normal University, Langfang, China

**Keywords:** *Nicrophorus nepalensis*, mitochondrial genome, Silphidae, phylogenetic analysis

## Abstract

The complete mitochondrial genome of burying beetle *Nicrophorus nepalensis* Hope, 1831 was reported in this study. It was 17,299 bps in length and included 13 protein-coding genes (PCGs), 22 tRNA genes, 2 rRNA genes, and a 2693 bp A + T-rich control region. Phylogenetic analysis using 13 PCGs from 38 Staphyliniform beetle species revealed that *N. nepalensis* was clustered in Silphidae, which together with Staphylinidae formed one monophyletic clade within Staphylinoidea.

*Nicrophorus nepalensis* Hope, 1831, a burying beetle species, belongs to the genus *Nicrophorus* Fabricius (Coleoptera: Silphidae). The adult beetles of *Nicrophorus* are renowned for their intriguing behavior of burying small vertebrate carcasses for food and sophisticated biparental care of the young (Scott 1998; Hwang and Lin 2013; Sikes and Venables 2013). The genus *Nicrophorus* currently consists of 68 species worldwide and is divided into 14 species groups or subgenera (Šípková and Růžička 2020). In *nepalensis* species group, *N. nepalensis* as the representative is the only eurytopic species that occurs across a broad elevation range in China, India, Japan, Pakistan, and much of the Oriental Region (Sikes et al. 2002; Mousseau and Sikes 2011; Šípková and Růžička 2020). The adult of *N. nepalensis* is about 20 mm in length; black in color; frons with a red-orange spot; clypeal membrane and apical 3 segments of antennae orange; elytra with anterior and posterior transverse fasciae orange-yellow, each fascia with a small black spot. The adult specimen used in this study was captured in 2019, from Guiyang Huaxi District (26°20′03′′N, 106°35′07′′E, 1100 m), Guizhou, China, using light trap. The specimen was immediately put in absolute alcohol after collection, and then stored in the Morphological Laboratory of Guizhou University of Traditional Chinese Medicine, Guiyang, China (Yanpeng Cai, cyp815@hotmail.com, Voucher specimen: GZUTCM:003).

The genome sequencing was performed on the Illumina HiSeq2500 platform, in Sangon Biotech (Shanghai) Co., Ltd., China. Software SPAdes V.3.14.1 (Bankevich et al. 2012) and MitoZ V.2.3 (Meng et al. 2019) were employed for the de novo assembly. Pilon V.1.23 (Walker et al. 2014) was used for sequence polish. The final annotation was carried out with the aid of both MitoZ software and MITOS Web Server (http://mitos2.bioinf.uni-leipzig.de/index.py).

The assembled mitogenome of *N. nepalensis* (GenBank accession number: MW365941) was a double-stranded circular DNA molecule, with 17,299 bps in size, and comprised 13 protein-coding genes (PCGs), 22 tRNA genes, and 2 rRNA genes, plus a putative control region (Wolstenholme 1992). All PCGs used conventional start codon (ATN), except for *cox1* and *nad1* which started with putative CCG and TTG, respectively. In terms of the stop codon, 6 PCGs (*atp6*, *atp8*, *nad2*, *nad4L*, *nad5*, and *nad6*) used TAA, 3 (*cytb*, *nad1*, and *nad3*) ended with TAG, while the rest 4 (*cox1*, *cox2*, *cox3*, and *nad4*) used single T as an incomplete stop codon. All tRNA genes, excluding TrnS1^AGN^ could fold into the iconic clover-leaf secondary structure. TrnS1^AGN^ formed a single-stranded loop instead of the DHU arm, and that the anticodon of trnS1^AGN^ in *N. nepalensis* was UCU rather than the more commonly used GCU. The overall base composition of *N. nepalensis* mitogenome was A 39.5%, T 37.2%, C 13.5%, and G 9.7%, with high AT content. The non-coding control region was 2,693 bp long, and strongly AT biased (AT 80.6%, CG 19.4%).

13 concatenated PCGs of *N. nepalensis* and other 37 Staphyliniform beetle species obtained from GenBank were used to build a ML phylogenetic tree via IQTREE V.2.07 (Nguyen et al. 2015; Figure 1). TESTMERGE option in IQTREE was selected to determine the best partition scheme. The dataset was finally divided and merged into 8 partitions, each applied with its own best fit substitution model and parameters (GTR + F + I + G4, TIM3 + F + I + G4, GTR + F + I + G4, GTR + F + I + G4, GTR + F + I + G4, GTR + F + I + G4, TPM3 + F + I + G4, TPM2 + F + G4). 1000 replicates of bootstrap analysis were executed to produce the nodal bootstrap values (NBV). The tree showed that 7 families with multiple sample species (Histeridae, Hydraenidae, Hydrochidae, Hydrophilidae, Leiodidae, Ptiliidae, Silphidae) were recovered as monophyla. Silphidae + Staphylinidae formed a monophyletic clade, which was consistent with a previous study (Mckenna et al. 2015). On the super family level, Hydrophiloidea was unexpectedly nested in Staphylinoidea with very weak support (NBV = 23), which conflicted with the conventional taxonomic cognition (Hydrophiloidea being a sibling to Histeroidea). Our *N. nepalensis* clustered in the family Silphidae with very strong support (NBV = 100).

**Figure 1.**
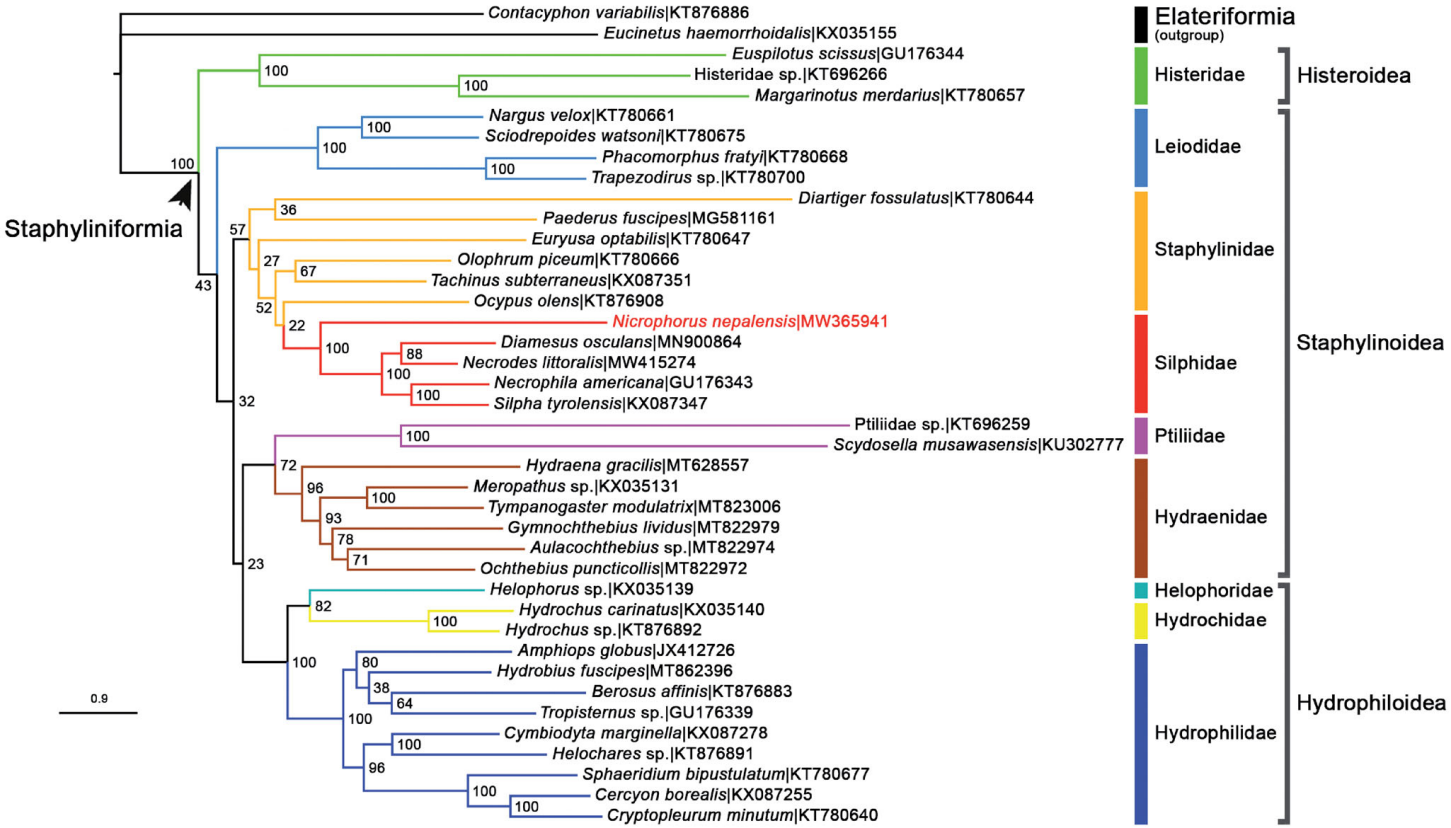
The ML phylogenetic tree was built from *N. nepalensis* (in red characters) and 37 other species from Staphyliniformia, with two Elateriform species selected as outgroup. Bootstrap support values were labeled at nodes. GenBank accession numbers of each species used in the study were also listed in the tree.

## Data Availability

The genome sequence data that support the findings of this study are openly available in GenBank of NCBI at https://www.ncbi.nlm.nih.gov/nuccore/MW365941 under the accession No. MW365941.
